# Membrane Fouling Mechanisms in the Microfiltration of Oat Protein–β-Glucan Complexes

**DOI:** 10.3390/membranes16040116

**Published:** 2026-03-27

**Authors:** Tianyu Zheng, Songlin Wen, Yi Wu, Pengyu Shuai, Delong Hou, Yao Jin

**Affiliations:** 1 College of Biomass Science and Engineering, Sichuan University, Chengdu 610065, China; 2023223080028@stu.scu.edu.cn (T.Z.); 2024323080023@stu.scu.edu.cn (S.W.); 2025223080007@stu.scu.edu.cn (Y.W.); 2025223080002@stu.scu.edu.cn (P.S.); 2 Key Laboratory for Leather and Engineering of the Education Ministry, Sichuan University, Chengdu 610065, China

**Keywords:** oat protein–β-glucan complexes, microfiltration, membrane fouling, pH effect, oat protein–β-glucan interactions

## Abstract

This work investigated the membrane fouling mechanisms during the microfiltration of oat protein–β-glucan complexes. Microfiltration experiments were conducted under various pH conditions, protein-to-polysaccharide ratios, and ionic strengths. The fouling behavior was analyzed using multiple membrane fouling models to systematically elucidate the relationships among the particle characteristics, rheological behaviors, and membrane fouling. When the pH was adjusted to 7.8, the multimodal particle size distribution of the complexes promoted the formation of a loosely structured cake layer on the membrane surface, accompanied by partial obstruction of membrane pore entrances. On the contrary, the complexes, shown as having a monomodal particle size distribution and similar particle size to the membrane pore, formed compact cake layers and strong membrane fouling resistance. At pH 4.8, protein hydrophobic aggregation generated large particulate clusters that formed a loose cake layer during microfiltration, resulting in a decrease in membrane fouling resistance. Increasing the β-glucan content reduced membrane resistance through enhancing steric hindrance and hydrophilicity. This research provides a theoretical foundation for optimizing membrane separation process parameters in the production of diversified oat-based products.

## 1. Introduction

In recent years, with the increasing global attention on healthy dietary patterns and environmental sustainability, plant proteins have garnered significant interest as potential alternatives to or complementary sources of animal proteins [1,2]. Oat, a highly nutritious crop, has emerged as a promising plant-based raw material due to its abundant content of functional polysaccharides and essential amino acids [3,4]. Oat protein exhibits favorable emulsifying and foaming properties, together with appropriate gelation characteristics [5], thereby conferring significant potential for applications in the food, nutrition, and health sectors.

Oat protein possesses an amphiphilic molecular structure that facilitates rapid adsorption at oil–water or air–water interfaces, thereby forming stabilizing films that maintain emulsion stability [6]. Furthermore, oat protein can form stable heat-induced gels by heating, enabling the manufacture of high-value food products such as plant-based yogurt, cheese, and meat analogs [7]. Oat grains are rich in β-glucan, which exhibits various physiological functions including cholesterol reduction and the promotion of intestinal digestion. Meanwhile, β-glucan serves as a dietary fiber fortifier and fat replacer, contributing to the improvement in food texture while conferring additional health benefits [8]. However, the high-value processing of oat grains faces considerable challenges, including high natural turbidity and poor protein solubility [9]. Membrane separation technology, characterized by its economic viability and ecological compatibility [10], represents a promising approach for oat processing applications.

Conventional oat processing typically involves grinding, enzymatic hydrolysis of polysaccharides, alkaline extraction followed by acid precipitation, and subsequent ultrafiltration concentration of proteins [11], followed by homogenization and sterilization. Nevertheless, the presence of microorganisms and lipids in oat protein precipitates compromises ultrafiltration membrane flux and diminishes the foaming properties of concentrated solutions [12]. Immonen et al. [13] employed organic solvents such as hexane to remove fats from ultrafiltration concentrates, achieving approximately 70% removal efficiency. Microfiltration, serving as a versatile separation technique for removing large particulate matter, reducing liquid turbidity, and concentrating target compounds [14], not only alleviates the burden on subsequent ultrafiltration processes but also offers economic and ecological advantages.

Interactions between protein and polysaccharides, including electrostatic and hydrophobic interactions as well as hydrogen bonding among others, can lead to distinct phase behaviors. Rodríguez et al. [15] explored the intermolecular interactions and phase behavior of protein–polysaccharide systems. At low concentrations, proteins and polysaccharides formed homogeneous mixtures in a single phase, a phenomenon known as thermodynamic compatibility. However, the system exhibited thermodynamic incompatibility at an elevated biopolymer concentration. Meanwhile, proteins and polysaccharides with different charge properties formed complexes via bridging flocculation. These systems exhibited either complexation, cosolubility or segregation. The intermolecular interactions within the complexes were closely associated with their microfiltration behavior. Lopez et al. [16] investigated the filtration performance and deposit shear stress of skim milk under various ionic conditions, characterizing the formation of irreversible deposits on membrane surfaces. At elevated ionic strengths, the ratio of flux to deposit shear stress decreased. The deposit structure became more consolidated, thereby increasing irreversible fouling resistance. However, studies specifically addressing membrane fouling mechanisms during the microfiltration of oat protein–β-glucan complexes remain scarce. Investigating membrane fouling mechanisms under different pH conditions and oat protein–β-glucan ratios is essential for elucidating the processing mechanisms of oat-based products such as oat yogurt, oat cheese, and neutral oat milk [17], with the aim of enhancing membrane separation efficiency.

Based on these considerations, this work investigated various microfiltration systems of oat protein–glucan complexes to explore fouling mechanisms under diverse conditions. The filtration performance, membrane fouling behavior, and the mechanisms of membrane pore blockage were evaluated and discussed, providing theoretical foundations for the industrial production of diverse oat-based products.

## 2. Materials and Methods

### 2.1. Preparation of the Oat Protein–β-Glucan Complex Solutions

Oat protein powder (hydrolyzed extraction, protein content: 98%, Shaanxi Dongjiang Kangtai Health Industry Co., Ltd., Xi’an, Shaanxi, China) and β-glucan (Cool Chemistry, Hefei, Anhui, China, MW ≈ 15 kDa) were utilized in this work. Oat protein powder was dissolved in 0.04 M sodium hydroxide solution with continuous stirring to prepare a 10 g/L oat protein stock solution. β-glucan was dissolved in deionized water to prepare a 10 g/L stock solution. All samples were stored overnight at 4 °C as precursors for preparing various complex solutions, with specific conditions detailed in Table 1.

### 2.2. Microfiltration System and Experimental Set-Up

The dead-end microfiltration apparatus employed in this work was consistent with the configuration described in our previous research [18]. Hydrophilic polyvinylidene fluoride (PVDF) membranes with 0.22 μm pore size (Kezhun Filtration Co., Ltd., Haining, Zhejiang, China.) were utilized. Before the filtration experiments, the membranes were pretreated by immersion in ultrapure water for 24 h. Compressed air was used to provide a constant transmembrane pressure of 0.02 MPa, and the stirring speed in the microfiltration cell was controlled at approximately 250 rpm. Filtrate was collected in a reservoir vessel, and filtrate mass was recorded every 5 s through the data acquisition system (SPDC, Version 2.01, Ohaus, Parsippany, NJ, USA). Each experiment was performed in triplicate to ensure accuracy.

### 2.3. Analysis of Membrane Fouling Mechanisms

#### 2.3.1. Resistance-in-Series Model

As previously reported [19], Darcy’s law was employed to calculate permeate flux during filtration and to evaluate the magnitude and distribution of resistance (Equation (1)):

(1) $$J\;=\frac{△P}{\mu R_{t}}$$

where *J* represents the flux of permeation (m^3^·m^−2^·s^−1^), Δ*P* denotes the transmembrane pressure (Pa), *μ* is the viscosity of filtrate (Pa·s), and *R_t_* corresponds to the total filtration resistance during the microfiltration process (m^−1^), which was calculated according to Equation (2).

(2) $$R_{t}=R_{m}+R_{f}={\;R}_{m}+R_{rf}+R_{irrf}=R_{m}+R_{rf}+R_{a}+R_{p}$$

*R_m_* is the intrinsic resistance of the virgin membrane (m^−1^), calculated according to Equation (3). The resistance of fouling layer (*R_f_*) generated during microfiltration is composed of reversible fouling resistance (*R_rf_*) and irreversible fouling resistance (*R_irrf_*) (Equations (4) and (5)). These parameters could be calculated by the next equations.

(3) $$\;\;R_{m}=\frac{△P}{\mu_{0}J_{0}}$$

(4) $$\;R_{f}=R_{t}-R_{m}=\frac{△P}{\mu J}-\frac{△P}{\mu_{0}J_{0}}$$

(5) $$\;\;\;R_{rf}=R_{f}-R_{irrf}=\frac{△P}{\mu J}-\frac{△P}{\mu_{0}J_{1}}$$

where *J*_0_ represents the pure water permeation flux of the virgin membrane (m^3^·m^−2^·s^−1^). *J*_1_ refers to the pure water flux of the contaminated membrane after cleaning. The viscosity of pure water is represented by *μ*_0_ (Pa·s).

#### 2.3.2. Fouling Propensity Model

The membrane fouling propensity model was employed to calculate the rate of membrane fouling growth and to evaluate differences in fouling behavior among various samples during filtration, as described in Equation (6):

(6) $$R_{t}\;=\;R_{m}\exp(K_{0}V/A)$$

where *V* refers to the filtration volume (m^3^), *A* represents the effective membrane area (m^2^), and *K*_0_ is the fouling coefficient (m^−1^), describing the rate of resistance growth during filtration. By rearranging and integrating Equations (7)–(9), the model can be expressed as Equation (10).

(7) $$J=\frac{1}{A}\frac{\mathrm{dV}}{\mathrm{dt}}=\frac{\Delta P}{\mu R_{t}}$$

(8) $$\frac{1}{A}\frac{\mathrm{dV}}{\mathrm{dt}\;}=\frac{\Delta P}{\mu R_{m}\exp(K_{0}V/A)}$$

(9) $$\int_{0}^{t}\mathrm{dt}=\frac{\mu R_{m}}{\Delta \mathrm{PA}}\int_{0}^{V}\exp(\frac{K_{0}V}{A})\mathrm{dV}$$

(10) $$V=\frac{A}{K_{0}}\ln(\frac{K_{0}\Delta P}{\mu R_{m}}t+1)$$

This model has been validated for membrane fouling analysis [20,21,22]. Experimental data were fitted using Equation (10) to calculate the fouling coefficient *K*_0_ under different conditions.

#### 2.3.3. Hermans and Bredee Model

Based on the approach proposed by Hermans and Bredee [23], the double logarithmic plot of *d*^2^*t/dv*^2^ versus *dt/dv*, derived from flux decline behavior, enables facile determination of membrane pore blocking mechanisms through linear regression fitting (Equation (11)):

(11) $$\frac{d^{2}t}{dv^{2}}\;=\;K{\left(\frac{\mathrm{dt}}{\mathrm{dv}}\right)}^{n}$$

where *K* is a constant and n is the blocking coefficient. Four models are typically employed for linear fitting. Generally, different n values indicate distinct membrane fouling mechanisms: 2.0 (complete blocking), 1.5 (standard blocking), 1.0 (intermediate blocking), and 0 (cake filtration). Integration of Equation (11) yields the following four linear equations:

For n = 2:

(12) $$J=J_{\mathrm{ini}}\exp(-K_{b}t)$$

For n = 1.5:

(13) $$J\;=\;\frac{J_{\mathrm{ini}}}{{\left(\frac{K_{s}J_{\mathrm{ini}}}{2}t+1\right)}^{2}}\;$$

For n = 1:

(14) $$J=J_{\mathrm{ini}}\exp\left(-K_{i}v\right)$$

For n = 0:

(15) $$\frac{t}{v}\;=\;\frac{K_{c}}{2}v\;+\;\frac{1}{J_{\mathrm{ini}}}$$

where *K_b_*, *K_s_*, *K_i_*, and *K_c_* are fouling model constants. Experimental data were fitted using Equations (12)–(15), and the regression coefficients were compared to determine the membrane pore blocking mechanism.

#### 2.3.4. Specific Cake Resistance and Fouling Layer Thickness Model

Specific cake resistance is a physical quantity measuring the intrinsic resistance of the fouling layer [24]. Fouling layer thickness represents the height of the physical layer formed by accumulated foulants on the membrane surface. The relationship between specific cake resistance and fouling layer thickness is described in Equation (16):

(16) $${\;R}_{c}=\int_{0}^{\omega }\alpha_{av}d\omega$$

Specific cake resistance *α_av_* is determined from cumulative permeate volume *V_cum_* [25,26], as described in the following Equation (17):

(17) $$\frac{t}{V_{cum}}\;=\;\alpha_{av}\times \frac{\eta \times \varphi_{OP-BG}}{2\times A_{M}^{2}\times △P}\times V_{cum}+\frac{\eta R_{M}}{A_{M}\times △P}$$

where *t* is the filtration time (s), *A_M_* is the effective filtration area of the membrane, *η* refers to the permeate viscosity, *ϕ* is the volume fraction of solids in the solution, and *R_M_* is the resistance of membrane.

### 2.4. Determination of Particle Properties

The particle size distribution, zeta potential, and polydispersity index (PDI) of the samples were analyzed using a Malvern Mastersizer instrument (ZEN3600 + MTP2, Malvern Panalytical, Malvern, UK). The particle size and PDI were obtained through dynamic light scattering (DLS), whereas zeta potential was evaluated using phase analysis light scattering (PALS).

### 2.5. Rheometric Measurements

The rheological behavior of oat protein–β-glucan complexes was measured using the rheometer (Anton Paar, MCR 302) at 25 °C. A cone-and-plate geometry was employed, equipped with a CP50-1 cone (50 mm diameter, 1° angle). During measurement, shear rate was increased from 0.1 to 300 s^−1^, and shear stress and shear rate were recorded.

If the fluid exhibited Newtonian characteristics or shear-thinning behavior without yield stress, the power-law model was employed for fitting:

(18) $$\tau \;=\;L\gamma^{m}$$

In this equation, *τ* is the shear stress (Pa), *L* is the viscosity coefficient (Pa·s^m^), *γ* is the shear rate (s^−1^), and *m* is the flow behavior index describing the shear-dependent flow characteristics of the fluid.

If the complex flow curve exhibited gel-like consistency and yield stress, the Herschel–Bulkley model was used for curve fitting [27]:

(19) $$\tau \;=\;\tau_{0}+L\gamma^{m}$$

where *τ*_0_ represents the yield stress (Pa).

### 2.6. Statistical Analysis

Each experiment was performed in triplicate to confirm reproducibility. The results were expressed as the mean value with the corresponding standard deviation. Data analysis was conducted using Origin 2025.

## 3. Results

### 3.1. Microfiltration Efficiency of Oat Protein–β-Glucan Complexes

**Figure 1:**
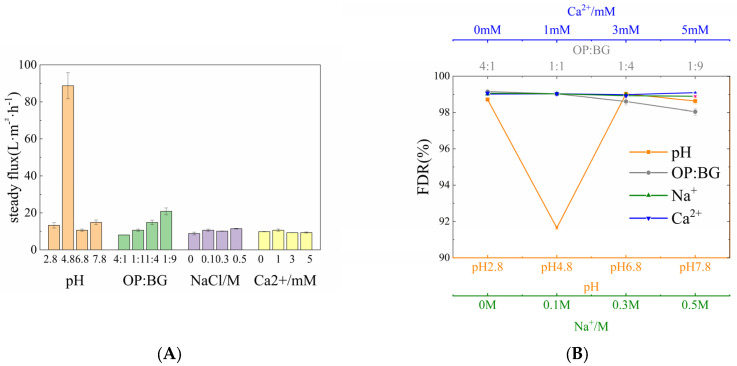
Effect of various solution conditions on the (**A**) steady permeate flux and (**B**) flux decline rate (FDR).

**Figure 2:**
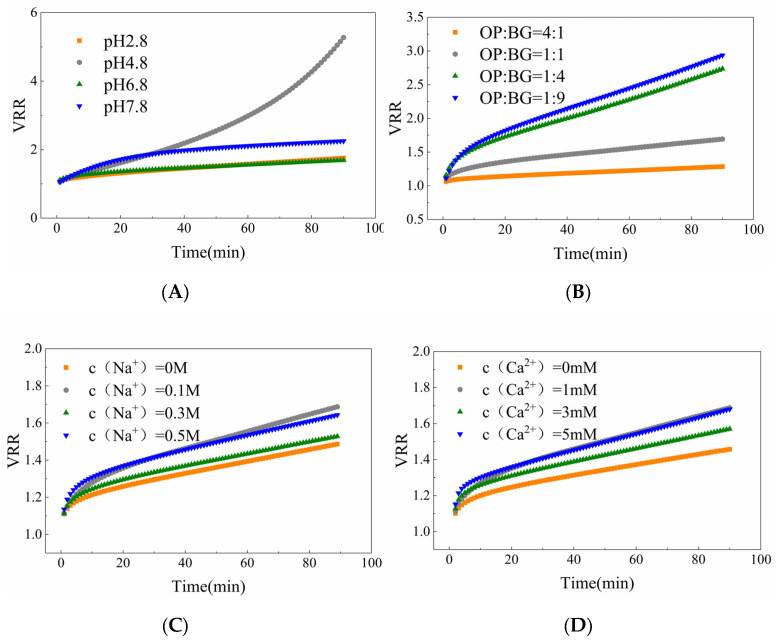
Effect of pH (**A**), oat protein–β-glucan ratios (**B**), Na^+^ concentrations (**C**), Ca^2+^ concentrations (**D**) on volume reduction ratio (VRR) of oat protein–β-glucan complexes microfiltration.

This section analyzed the filtration efficiency of oat protein–β-glucan complexes under different conditions. As shown in Figure 1A, pH was a significant factor in affecting membrane flux. Increasing the concentration of β-glucan contributed to a slight increase in steady-state membrane flux, while the steady-state membrane flux remained essentially unchanged across different NaCl and CaCl_2_ concentrations. As illustrated in Figure 1B, the flux decline rate (FDR) was significantly lower at pH 4.8 compared to those under other conditions, whereas the ratios (OP-BG), c (NaCl) and c (Ca^2+^) were not primary factors affecting the FDR. The volume reduction ratio (VRR) shown in Figure 2 revealed filtration efficiency under different conditions. At pH 4.8, filtration efficiency increased rapidly, while the resistance increased relatively slowly under other conditions. With the increase in β-glucan concentration, the VRR increased more gradually.

### 3.2. Characteristics of Oat Protein–β-Glucan Complexes

#### 3.2.1. Particle Characteristics

**Figure 3:**
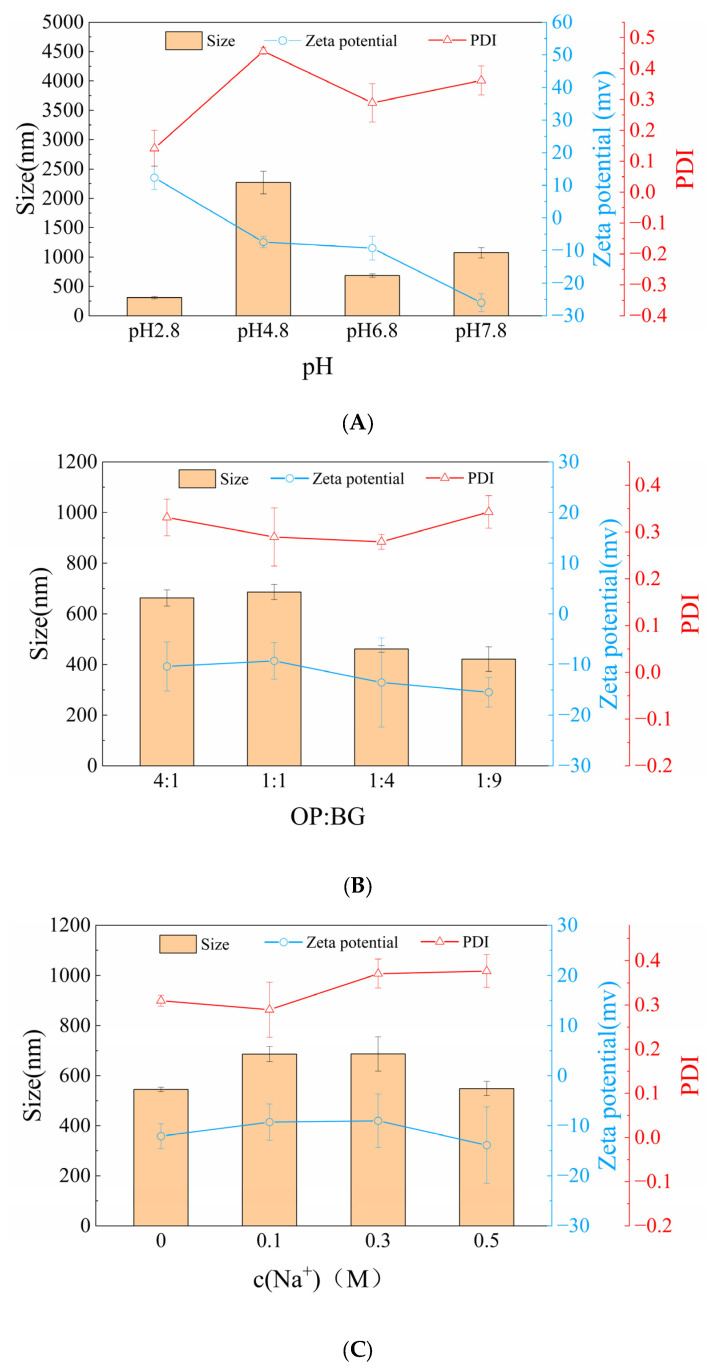

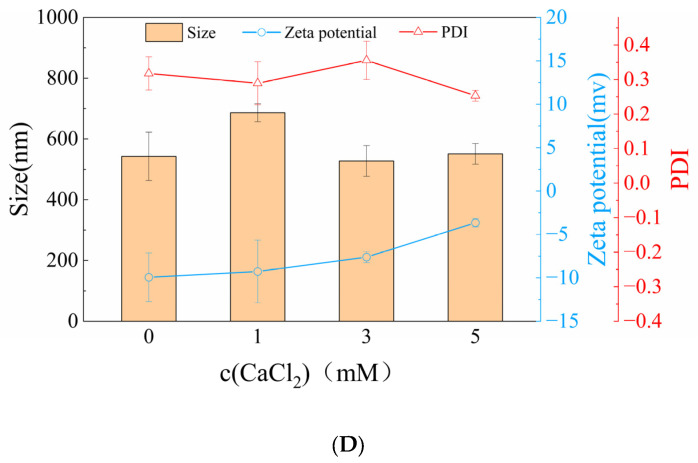
The particle properties (size, zeta potential and PDI) of oat protein–β-glucan under various pH (**A**), oat protein–β-glucan ratios (**B**), Na^+^ concentrations (**C**), Ca^2+^ concentrations (**D**).

Figure 3 illustrates the variations in oat protein–β-glucan complex particle size and zeta potential under various solution conditions. The pH was the dominant factor affecting solution properties. At pH 2.8, the complex exhibited minimal particle size, close to the membrane pore size. These particles were adsorbed within the membrane pores, resulting in irreversible membrane fouling during microfiltration [28]. The isoelectric point of oat protein was 4–5 [29]. Protein aggregation and precipitation occurred, leading to the formation of larger aggregated particles. Compared to the solution at pH≈pI, the complex particle size under the condition of pH > pI was smaller. Figure 3B demonstrates the particle characteristics of solutions under different ratios of OP-BG. It indicates that the particle size was larger when OP:BG ≥ 1:1.

As shown in Figure 3, the pH was the primary factor affecting the zeta potential of the complexes. The zeta potential gradually decreased with increasing pH. At pH 4.8 and 6.8, the |zeta| was relatively small, indicating lower stability of the complexes. Figure 3B–D indicate that zeta potential did not change significantly with variations in OP-BG ratios, c (NaCl) and c (Ca^2+^).

#### 3.2.2. Flow Properties of Oat Protein–β-Glucan Complexes

Oat protein–β-glucan complex solutions exhibited specific flow and deformation responses under external forces, thereby revealing their viscosity, gel properties and phase behavior [30]. Typically, stable oat protein–β-glucan complexes formed a tight gel network, with minimal phase separation between phases. Previous studies had investigated the rheological properties of β-glucan derived from oats [31], whereas rheological studies on oat protein–β-glucan complexes remain scarce.

**Figure 4:**
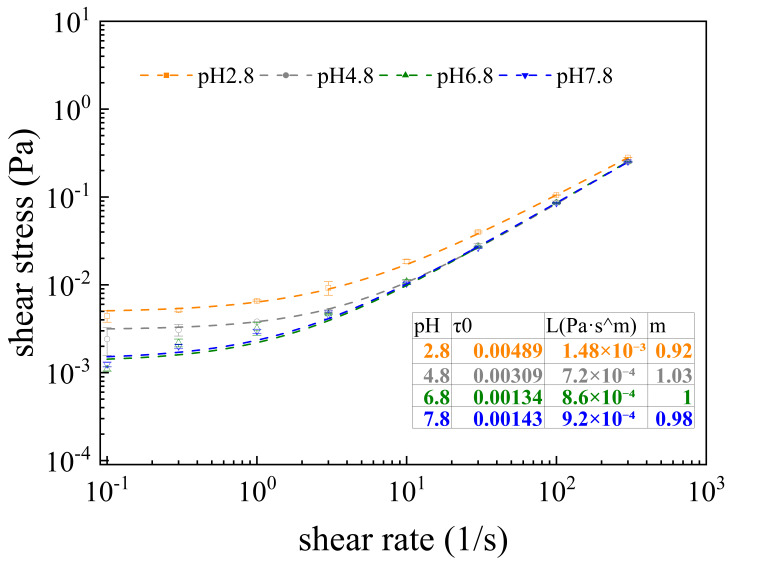
Rheological curves of oat protein–β-glucan complexes at pH 2.8, 4.8, 6.8, 7.8.

This section evaluates the flow characteristics of oat protein–β-glucan complexes through rheometric measurements. Figure 4 presents the rheological behaviors of oat protein–β-glucan complexes under various pH conditions. The flow properties of the complexes were classified into two categories based on pH. Firstly, at pH 4.8, 6.8, and 7.8, the m values of the complexes were close to 1, suggesting that the systems behaved as Newtonian fluids. Particularly, at pH 2.8, the complex exhibited the lowest m value among all pH conditions, displaying shear-thinning behavior.

### 3.3. Membrane Fouling Behavior of Oat Protein–β-Glucan Complexes

#### 3.3.1. Distribution of Membrane Fouling Resistance

**Figure 5:**
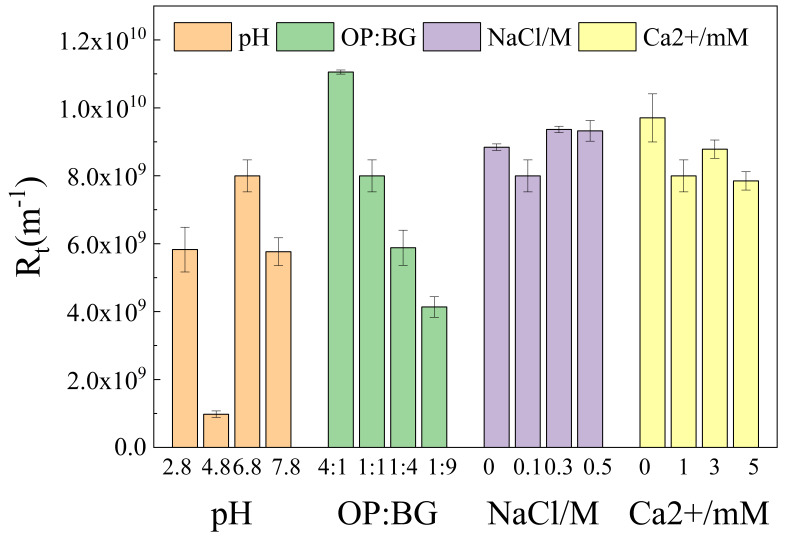
The total resistance of oat protein–β-glucan complexes in various conditions.

**Figure 6:**
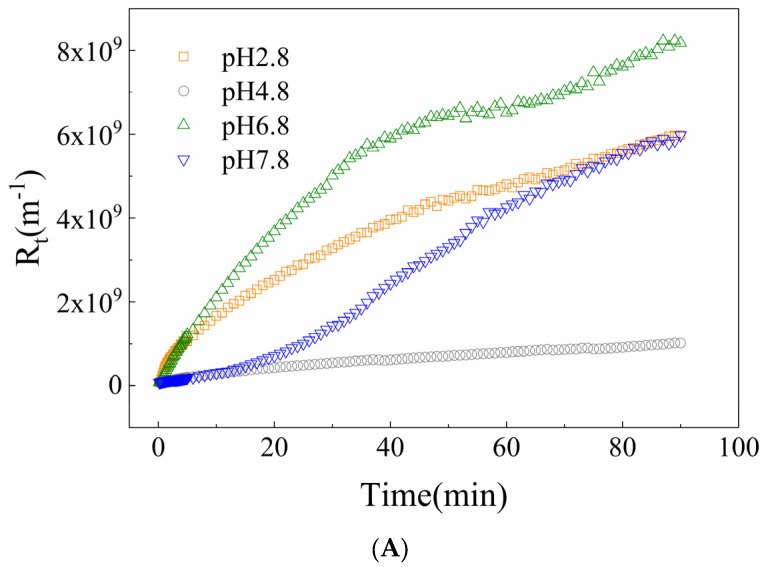

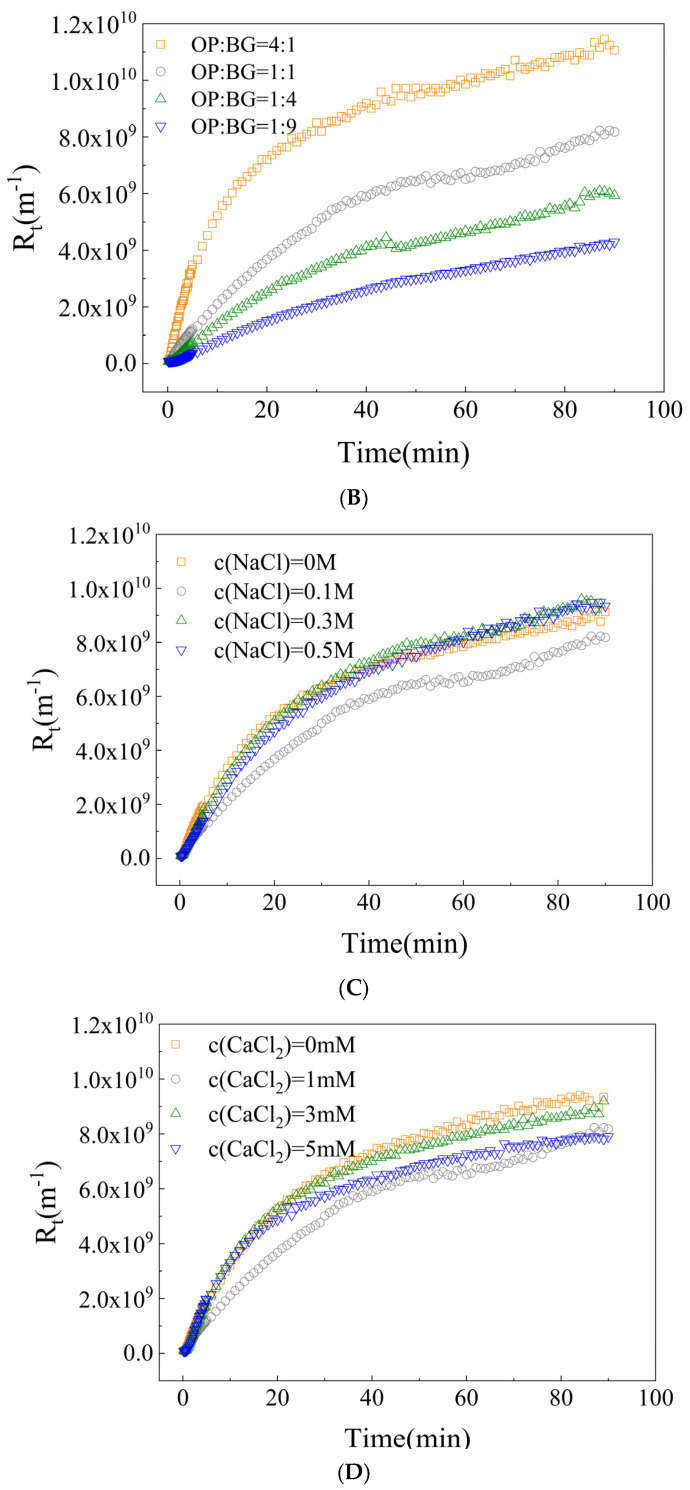
The trend of total resistance of oat protein–β-glucan complexes microfiltration under various pH (**A**), oat protein–β-glucan ratios (**B**), Na^+^ concentrations (**C**), Ca^2+^ concentrations (**D**).

**Figure 7:**
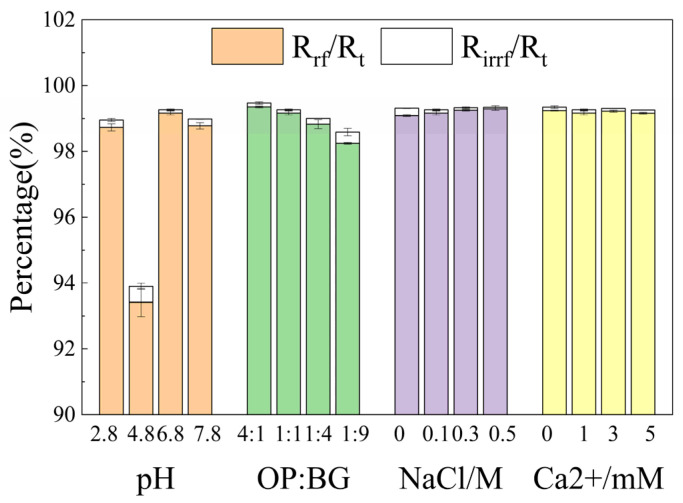
The percentage of reversible resistance (*R_rf_*) and irreversible resistance (*R_irrf_*) in total resistance (*R_t_*) in various conditions.

Figure 5 and Figure 6 illustrate the total membrane fouling resistance and its tendencies during microfiltration, respectively. Figure 7 shows the percentages of reversible and irreversible fouling resistance. At pH 4.8, the total resistance of oat protein–β-glucan complexes was the lowest among all pH conditions, with the highest proportion of irreversible fouling resistance. The highest total resistance occurred at pH 6.8. In the process of microfiltration, the total resistance under the pH 4.8 condition grew steadily, while membrane fouling under other conditions continued to worsen. The ratios of OP-BG also had a significant impact on the microfiltration resistance. Generally, an increase in β-glucan concentration generally led to a reduction in the total resistance and its rate of increase.

#### 3.3.2. Fouling Propensity of Oat Protein–β-Glucan Complexes

**Figure 8:**
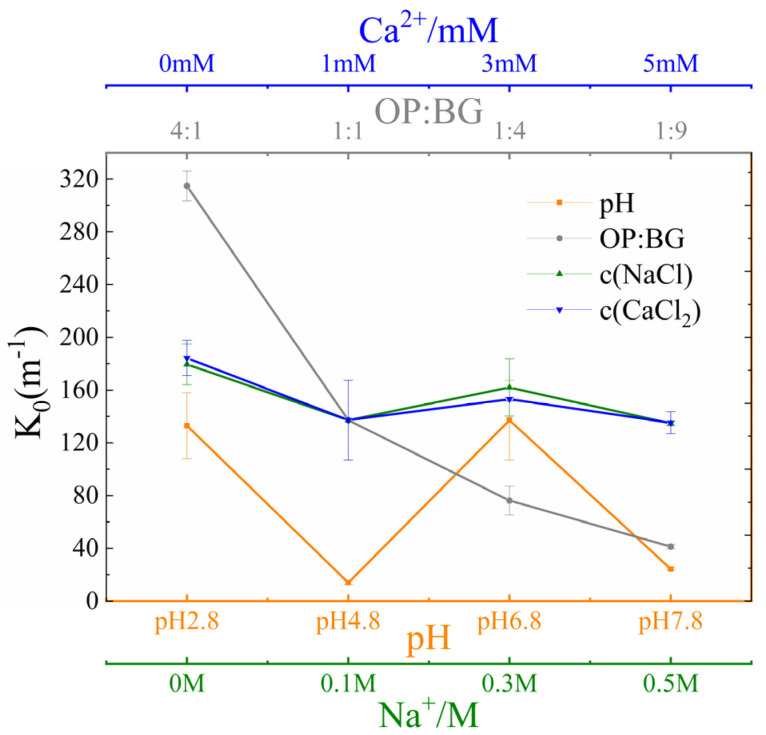
Fouling coefficient (*K*_0_) of oat protein–β-glucan complexes in diverse conditions.

The fouling propensity coefficient *K*_0_ provided an important basis and model foundation for accurately predicting membrane fouling behavior, which was beneficial to optimize filtration operating solution conditions [32]. The fouling propensity (*K*_0_) of the complexes exhibited different characteristics under various conditions (Figure 8). At pH 2.8 and 6.8, membrane fouling propensity was higher than that at pH 4.8 and 7.8. Additionally, a higher β-glucan concentration led to a slower development of membrane fouling. Regarding c (NaCl) and c (Ca^2+^), neither exhibited significant effects on *K*_0_.

#### 3.3.3. Specific Cake Resistance and Fouling Layer Thickness

Specific cake resistance (*α_av_*) referred to the resistance to fluid microfiltration per unit fouling layer, uncovering the compactness of the fouling layer structure. Fouling layer thickness (*ω*) was involved with the thickness of the cake layer generated by particles accumulated on the membrane surface during microfiltration, intuitively reflecting the accumulation of foulants [33].

**Figure 9:**
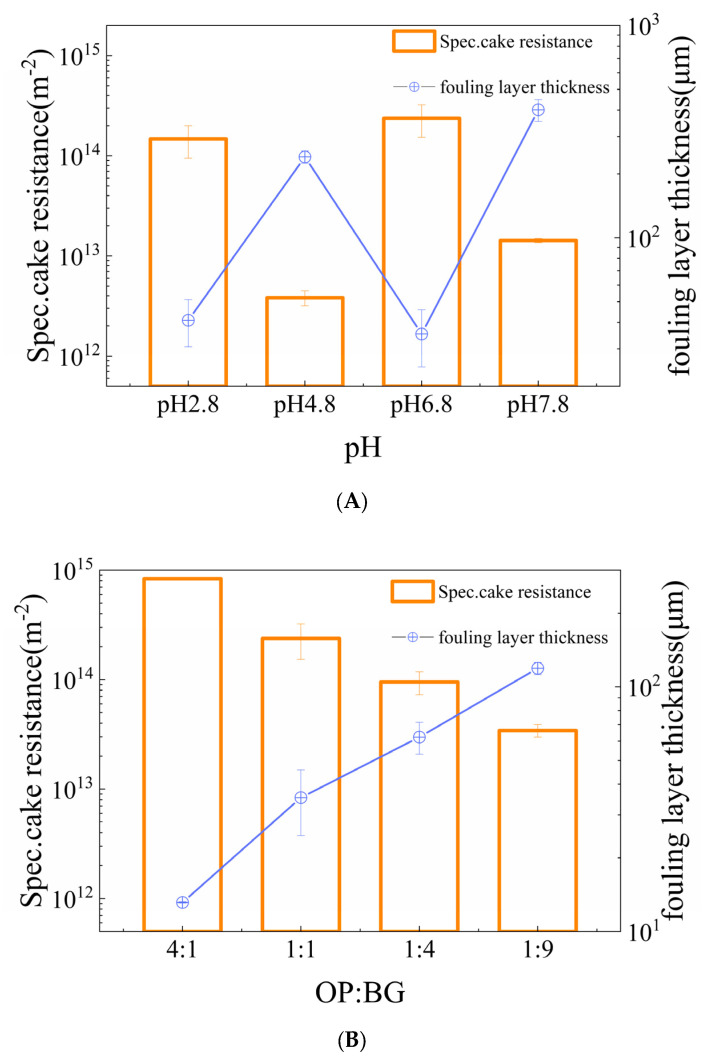

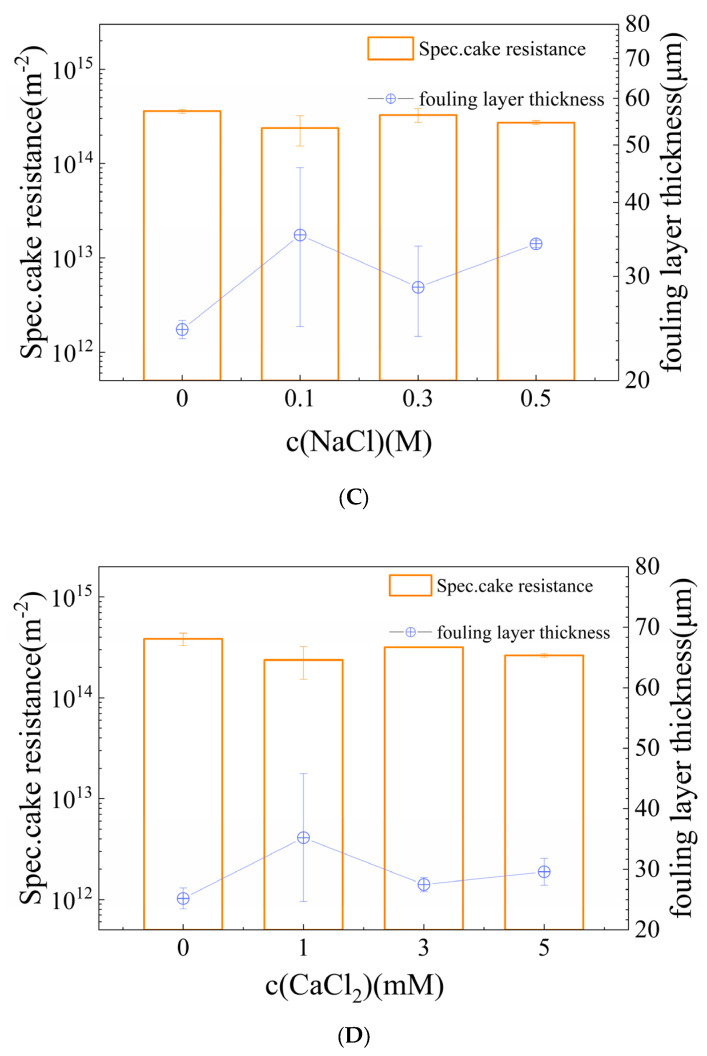
The specific cake resistance (*α_av_*) and fouling layer thickness (*ω*) of oat protein–β-glucan complexes during microfiltration in various pH (**A**), oat protein–β-glucan ratios (**B**), Na^+^ concentrations (**C**), Ca^2+^ concentrations (**D**).

Figure 9A indicates that, compared to pH 4.8 and 7.8 conditions, specific fouling resistance (*α_av_*) at pH 2.8 and 6.8 increased dramatically along with the thin deposit layer solid height (*ω*). Figure 9B shows that, as the ratios (OP-BG) declined from 4:1 to 1:9, specific cake resistance (*α_av_*) decreased and the fouling layer thickness (*ω*) gradually thickened. Figure 9C–D demonstrate that the presence or absence of Na^+^ and Ca^2+^ did not have significant effects on specific fouling resistance (*α_av_*).

#### 3.3.4. Pore Blocking Mechanism

**Figure 10:**
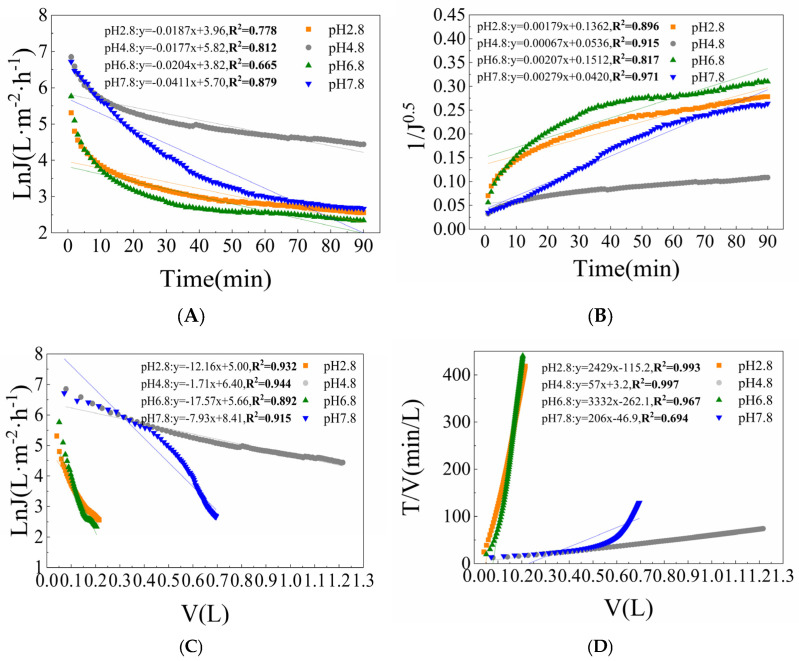
Pore blocking model fitting of oat protein–β-glucan complexes microfiltration at pH 2.8, 4.8, 6.8, 7.8. (**A**–**D**) represent complete blocking, standard blocking, intermediate blocking and cake filtration respectively.

The microfiltration membrane fouling behaviors of oat protein–β-glucan complexes depended on the interactions between the complexes and membrane pores. This section employed the Hermans and Bredee model to analyze the microfiltration fouling mechanisms of the complexes. Figure 10 presents fitting results for the microfiltration behaviors of complex solutions at four pH values. At pH 2.8, 4.8, and 6.8, the filtration data were well fitted by intermediate blocking and cake filtration models. However, at pH 7.8, the membrane fouling resistance was dominated by standard blocking and intermediate blocking, implying an internal pore blocking. Cake filtration was identified as the primary fouling mechanism under all other conditions (Figures S4–S6).

## 4. Discussion

### 4.1. Effect of pH on Membrane Fouling

Based on the above results, pH variation had a greater impact on microfiltration flux and resistance than the ratio (OP-BG), c(Na^+^) and c(Ca^2+^). Therefore, the following analysis focuses on the particle, rheological characteristics and pore blocking mechanisms of oat protein–β-glucan complexes under different pH conditions to explain microfiltration behaviors.

#### 4.1.1. pH ≈ pI

At pH 4.8, oat protein carried nearly zero net charge. Consequently, electrostatic repulsion between protein and glucan was minimized, while the exposure of hydrophobic groups in oat protein promoted complex aggregation to form large particulate clusters. Figure 4 demonstrates that the complex exhibits Newtonian flow behavior under the pH 4.8 condition. As shown in Figure 3, the particle size of the complexes at pH 4.8 was significantly larger than the pore size of the microfiltration membrane (0.22 μm), indicating that the complex particles were less likely to be adsorbed within membrane pores during microfiltration. The complex exhibiting maximum particle size and relatively low apparent viscosity formed porous cake layers with high permeability during microfiltration [34,35]. This was manifested as the minimal rate of membrane fouling and specific cake resistance. The particle size and rheological characteristics of the complexes may also explain why the filtration efficiency (VRR) at pH 4.8 was significantly higher than that under other pH conditions (Figure 2). This observation is consistent with the results reported by Wen et al. [36], who investigated the microfiltration behavior of casein–chitosan complexes under different solution conditions. In their study, the complexes at pH 6.8 exhibited the largest particle size and the lowest apparent viscosity among the four pH conditions, which led to the formation of a loose and highly permeable cake layer during microfiltration, thereby enhancing permeate flux and improving filtration efficiency.

#### 4.1.2. pH < pI or pH > pI

Compared to pH 4.8, the complex solutions at pH 2.8, 6.8, and 7.8 exhibited a significant growth of total resistance during microfiltration. This phenomenon might be attributed to electrostatic repulsion and hydrophobic interactions among the complexes, as well as interactions between complexes and the membrane. At pH 2.8, electrostatic repulsion prevented protein aggregation and led to minimal particle size [37], which was close to the membrane pore dimensions. Simultaneously, the hydrogen bonds promoted the crosslinking of oat proteins and β-glucan [38], which generated maximum apparent viscosity and the most compact gel structure (Figure 4). In the process of microfiltration, the complexes formed dense gel layers on the membrane surface, while particles permeated into and were adsorbed within the membrane pores, creating the highest irreversible fouling resistance among all pH conditions [39].

Under the pH 6.8 and 7.8 conditions, low permeate flux was similarly observed, which might be mainly attributed to interactions between complexes and the microfiltration membrane. When pH > pI, protein conformation became relatively extended, protein solubility increased, and hydrophobic interactions weakened [40]. Figure 3 indicates that the complexes under these conditions exhibited significantly larger particle sizes compared to pH 2.8, while Figure 4 demonstrates negligible gel characteristics. Large and loose complexes typically exhibited high permeate flux during microfiltration [35]. The contradictory results might be attributed to interactions between the complexes and the PVDF membrane. Chiao et al. [41] investigated particle–membrane interactions during ultrafiltration and found that proteins could be adsorbed onto the membrane surface through hydrogen bonding and electrostatic interactions. Zhi et al. [42] incorporated cellulose into PVDF matrices. Through membrane structure characterization, hydrogen bonding was identified between nanofibers and PVDF. Oat proteins contained abundant polar groups such as carboxyl and amino groups, while hydrophilic PVDF membranes possessed polar groups, including sulfonic groups on the surfaces. At pH 6.8 and 7.8, the polar groups of oat proteins were exposed, which enhanced their interactions with the hydrophilic PVDF membrane. These interactions mainly involved hydrogen bonding, electrostatic attraction and other intermolecular forces. The combined effects of these interactions promoted the adsorption and deposition of particles on the membrane surface, thereby aggravating membrane fouling.

**Figure 11:**
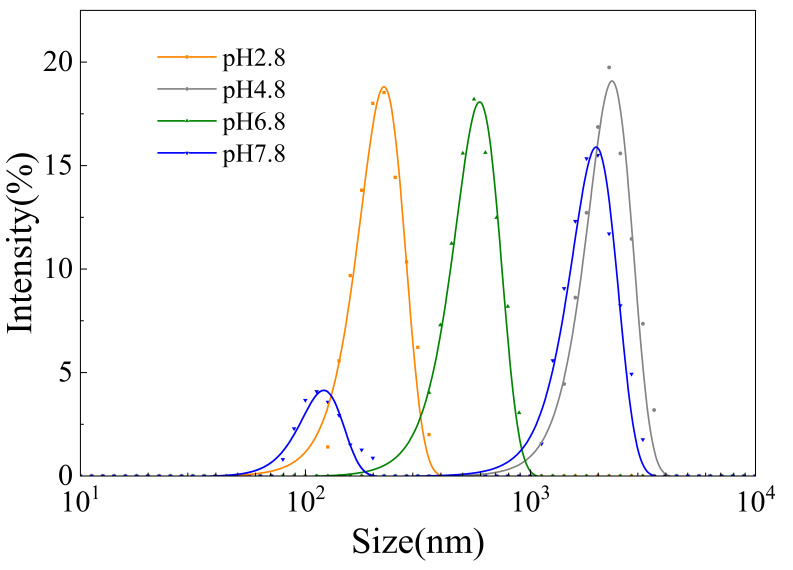
Particle size distributions of oat protein–β-glucan complexes under various pH conditions.

Notably, unlike microfiltration mechanisms at other pH conditions, cake fouling was not the primary membrane fouling mechanism for the complex microfiltration at pH 7.8. Conversely, standard blocking and intermediate blocking predominated. Pore blocking mechanisms were determined by particles and membrane behaviors [43,44]. Figure 11 shows the particle size distributions of oat protein–β-glucan complexes under various pH conditions. At pH 7.8, the particle size was several times larger than the membrane pore size. However, multimodal particle distribution was observed. Inhomogeneous particle distribution was unfavorable for forming multilayer structures, which is the typical cake filtration mechanism [45]. Meanwhile, some small particles blocked membrane pore entrances or penetrated into membrane pores, leading to the standard blocking and intermediate blocking.

Interestingly, the membrane fouling propensity at pH 7.8 was considerably lower than that at pH 2.8 and pH 6.8, which appeared to contradict the results of total membrane fouling resistance. Low fouling propensity typically indicated that foulants were less prone to deposit on the membrane surface [46]. However, under pH 7.8 conditions, cake filtration was not the predominant characteristic of membrane fouling. Instead, small particles were adsorbed into the internal membrane pores, generating substantial pore blocking resistance (*R_p_*), thereby increasing the total membrane fouling resistance.

### 4.2. Effects of Oat Protein–β-Glucan Ratio and Ionic Strength on Membrane Fouling

For different ratios (OP-BG), resistance declined with increasing β-glucan content (Figure 5), accompanied by improved filtration efficiency (Figure 2), consistent with previous reports [36]. Glucan chains are highly hydrophilic. As the content of β-glucan increases, the molecular hydration layers and steric hindrance thicken, effectively inhibiting hydrophobic aggregation and precipitation, thereby reducing the tendency to form dense fouling layers on membrane surfaces [47], which affects the volume reduction ratio (VRR) of the system. A more porous and permeable deposited layer was formed with the increase in β-glucan content, resulting in a slower flux decline during microfiltration. This allowed more permeate to be removed during the filtration process, which was reflected by the increased VRR.

By adding NaCl to the solution, the membrane fouling resistance reached the minimum level in the basic condition. The low concentration of Na^+^ decreased membrane fouling resistance caused by the reduction in repulsion between protein molecules, which promoted the formation of large and loose aggregates. However, at higher Na^+^ concentrations, strong electrostatic shielding effects nearly eliminated electrostatic repulsion, leading to hydrophobic aggregation of oat protein–β-glucan complexes, forming relatively dense fouling layers that increased membrane resistance [48].

## 5. Conclusions

This work elucidated the membrane fouling mechanisms of oat protein–β-glucan complexes from the perspectives of particle characteristics and flow properties. The results indicated that membrane fouling behavior was primarily influenced by pH and ratios (OP-BG). Increasing the β-glucan content in the complexes reduced membrane resistance, while Na^+^ and Ca^2+^ had relatively insignificant effects on membrane resistance. pH determined membrane fouling resistance and mechanisms by influencing particle and rheological properties. At pH 4.8, the total resistance was lowest. At pH 7.8, fouling uniquely exhibited standard blocking and intermediate blocking, whereas cake filtration predominated under other pH conditions. Practically, investigations of membrane fouling mechanisms in oat protein–β-glucan complex systems under diverse conditions provide theoretical foundations for membrane fouling control in the production of various oat-based products.

## Figures and Tables

**Table 1:** Solution conditions of experimental samples (oat protein–β-glucan complexes).

Experimental Parameters Under the Solution Conditions	Standard Condition	Additional Conditions
pH	6.8	2.8, 4.8, 7.8
oat protein: β-glucan	1:1	4:1, 1:4, 1:9
c (NaCl)	0.1 M	0 M, 0.3 M, 0.5 M
c (Ca^2+^)	1 mM	0 mM, 3 mM, 5 mM

Note: total concentrations of oat protein–β-glucan complexes were all 1 g/L.

## Data Availability

The original contributions presented in this study are included in the article/Supplementary Material. Further inquiries can be directed to the corresponding authors.

## Supplementary Materials

The following supporting information can be downloaded at: https://www.mdpi.com/article/10.3390/membranes16040116/s1, Figure S1: Rheological curves of oat protein–β-glucan complexes in the conditions of different oat protein–β-glucan ratios; Figure S2: Rheological curves of oat protein–β-glucan complexes in the conditions of different sodium ion concentrations; Figure S3: Rheological curves of oat protein–β-glucan complexes in the conditions of different calcium ion concentrations; Figure S4: Pore blocking model fitting of oat protein–β-glucan complexes microfiltration with different oat protein–β-glucan ratios. (A–D) represent complete blocking, standard blocking, intermediate blocking and cake filtration respectively; Figure S5: Pore blocking model fitting of oat protein–β-glucan complexes microfiltration with different sodium ion concentrations. (A–D) represent complete blocking, standard blocking, intermediate blocking and cake filtration respectively; Figure S6: Pore blocking model fitting of oat protein–β-glucan complexes microfiltration with different calcium ion concentrations. (A–D) represent complete blocking, standard blocking, intermediate blocking and cake filtration respectively.

## References

[1] Nikbakht Nasrabadi M., Sedaghat Doost A., Mezzenga R. (2021). Modification Approaches of Plant-Based Proteins to Improve Their Techno-Functionality and Use in Food Products. Food Hydrocoll..

[2] Poore J., Nemecek T. (2018). Reducing Food’s Environmental Impacts through Producers and Consumers. Science.

[3] Nguyen T.T.L., Gilbert R.G., Gidley M.J., Fox G.P. (2020). The Contribution of β-Glucan and Starch Fine Structure to Texture of Oat-Fortified Wheat Noodles. Food Chem..

[4] Sánchez-Velázquez O.A., Cuevas-Rodríguez E.O., Mondor M., Ribéreau S., Arcand Y., Mackie A., Hernández-Álvarez A.J. (2021). Impact of in Vitro Gastrointestinal Digestion on Peptide Profile and Bioactivity of Cooked and Non-Cooked Oat Protein Concentrates. Curr. Res. Food Sci..

[5] Boukid F. (2021). Oat Proteins as Emerging Ingredients for Food Formulation: Where We Stand?. Eur. Food Res. Technol..

[6] Wang X., Lei Y., Rafique H., Zou L., Hu X. (2023). Effect of Stir-Frying on Physicochemical and Functional Properties of Oat Protein Isolates. Foods.

[7] Mel R., Rampitsch C., Zvomuya F., Nilsen K.T., Beattie A.D., Malalgoda M. (2024). Determining the Impact of Genotype × Environment on Oat Protein Isolate Composition Using HPLC and LC-MS Techniques. J. Agric. Food Chem..

[8] Kaur R., Sharma M., Ji D., Xu M., Agyei D. (2019). Structural Features, Modification, and Functionalities of Beta-Glucan. Fibers.

[9] Brückner-Gühmann M., Benthin A., Drusch S. (2019). Enrichment of Yoghurt with Oat Protein Fractions: Structure Formation, Textural Properties and Sensory Evaluation. Food Hydrocoll..

[10] Kamali M., Suhas D.P., Costa M.E., Capela I., Aminabhavi T.M. (2019). Sustainability Considerations in Membrane-Based Technologies for Industrial Effluents Treatment. Chem. Eng. J..

[11] Immonen M., Myllyviita J., Sontag-Strohm T., Myllärinen P. (2021). Oat Protein Concentrates with Improved Solubility Produced by an Enzyme-Aided Ultrafiltration Extraction Method. Foods.

[12] Yue J., Gu Z., Zhu Z., Yi J., Ohm J.-B., Chen B., Rao J. (2021). Impact of Defatting Treatment and Oat Varieties on Structural, Functional Properties, and Aromatic Profile of Oat Protein. Food Hydrocoll..

[13] Immonen M., Chandrakusuma A., Hokkanen S., Partanen R., Mäkelä-Salmi N., Myllärinen P. (2022). The Effect of Deamidation and Lipids on the Interfacial and Foaming Properties of Ultrafiltered Oat Protein Concentrates. LWT.

[14] El Rayess Y., Albasi C., Bacchin P., Taillandier P., Mietton-Peuchot M., Devatine A. (2012). Analysis of Membrane Fouling during Cross-Flow Microfiltration of Wine. Innov. Food Sci. Emerg. Technol..

[15] Rodríguez Patino J.M., Pilosof A.M.R. (2011). Protein–Polysaccharide Interactions at Fluid Interfaces. Food Hydrocoll..

[16] Jimenez-Lopez A.J.E., Leconte N., Garnier-Lambrouin F., Bouchoux A., Rousseau F., Gésan-Guiziou G. (2011). Ionic Strength Dependence of Skimmed Milk Microfiltration: Relations between Filtration Performance, Deposit Layer Characteristics and Colloidal Properties of Casein Micelles. J. Membr. Sci..

[17] Yu Y., Li X., Zhang J., Li X., Wang J., Sun B. (2023). Oat Milk Analogue versus Traditional Milk: Comprehensive Evaluation of Scientific Evidence for Processing Techniques and Health Effects. Food Chem. X.

[18] Guo H., Li Z., Huang J., Zhou R., Wu C., Jin Y. (2020). Microfiltration of Soy Sauce: Efficiency, Resistance and Fouling Mechanism at Different Operating Stages. Sep. Purif. Technol..

[19] Piry A., Heino A., Kühnl W., Grein T., Ripperger S., Kulozik U. (2012). Effect of Membrane Length, Membrane Resistance, and Filtration Conditions on the Fractionation of Milk Proteins by Microfiltration. J. Dairy Sci..

[20] Zhu Z., Luo X., Yin F., Li S., He J. (2018). Clarification of Jerusalem Artichoke Extract Using Ultra-Filtration: Effect of Membrane Pore Size and Operation Conditions. Food Bioprocess Technol..

[21] Wan Y., Prudente A., Sathivel S. (2012). Purification of Soluble Rice Bran Fiber Using Ultrafiltration Technology. LWT—Food Sci. Technol..

[22] Zhu Z., Luo J., Ding L., Bals O., Jaffrin M.Y., Vorobiev E. (2013). Chicory Juice Clarification by Membrane Filtration Using Rotating Disk Module. J. Food Eng..

[23] Sousa M.R.S., Lora-Garcia J., López-Pérez M.-F. (2018). Modelling Approach to an Ultrafiltration Process for the Removal of Dissolved and Colloidal Substances from Treated Wastewater for Reuse in Recycled Paper Manufacturing. J. Water Process Eng..

[24] Dilaver M., Soydemir G., Dursun M., Murat Hocaoğlu S., Keskinler B., Ağtaş M., Koyuncu İ., Alp K. (2023). Highly Alkali Caustic Discharges Recovery Using Tubular and Disc Type of Ceramic Membranes and Its Applicability as a near Zero Liquid Discharge Opportunity in the Textile Industry. J. Environ. Chem. Eng..

[25] Steinhauer T., Lonfat J., Hager I., Gebhardt R., Kulozik U. (2015). Effect of pH, Transmembrane Pressure and Whey Proteins on the Properties of Casein Micelle Deposit Layers. J. Membr. Sci..

[26] Steinhauer T., Hanély S., Bogendörfer K., Kulozik U. (2015). Temperature Dependent Membrane Fouling during Filtration of Whey and Whey Proteins. J. Membr. Sci..

[27] Jin Y., Hengl N., Baup S., Pignon F., Gondrexon N., Sztucki M., Gésan-Guiziou G., Magnin A., Abyan M., Karrouch M. (2014). Effects of Ultrasound on Cross-Flow Ultrafiltration of Skim Milk: Characterization from Macro-Scale to Nano-Scale. J. Membr. Sci..

[28] Trzaskus K., Elshof M., Kemperman A., Nijmeijer K. (2016). Understanding the Role of Nanoparticle Size and Polydispersity in Fouling Development during Dead-End Microfiltration. J. Membr. Sci..

[29] Wang J., Yang C., Jiang J. (2025). Pickering Emulsions with Controllable Rheological Properties Stabilized by Oat Globulin at Different pH Levels. Colloids Surf. A Physicochem. Eng. Asp..

[30] Oates K.M.N., Krause W.E., Jones R.L., Colby R.H. (2006). Rheopexy of Synovial Fluid and Protein Aggregation. J. R. Soc. Interface.

[31] Dongowski G., Drzikova B., Senge B., Blochwitz R., Gebhardt E., Habel A. (2005). Rheological Behaviour of β-Glucan Preparations from Oat Products. Food Chem..

[32] Sandoval-García V., Ruano M.V., Alliet M., Brepols C., Comas J., Harmand J., Heran M., Mannina G., Rodriguez-Roda I., Smets I. (2025). Modeling MBR Fouling: A Critical Review Analysis towards Establishing a Framework for Good Modeling Practices. Water Res..

[33] Mahamadou Harouna B., Benkortbi O., Hanini S., Amrane A. (2019). Modeling of Transitional Pore Blockage to Cake Filtration and Modified Fouling Index—Dynamical Surface Phenomena in Membrane Filtration. Chem. Eng. Sci..

[34] Zator M., Ferrando M., López F., Güell C. (2009). Microfiltration of Protein/Dextran/Polyphenol Solutions: Characterization of Fouling and Chemical Cleaning Efficiency Using Confocal Microscopy. J. Membr. Sci..

[35] Castaing J.B., Massé A., Séchet V., Sabiri N.-E., Pontié M., Haure J., Jaouen P. (2011). Immersed Hollow Fibres Microfiltration (MF) for Removing Undesirable Micro-Algae and Protecting Semi-Closed Aquaculture Basins. Desalination.

[36] Wen S., Huang J., Zhou R., Wu C., Hengl N., Pignon F., Jin Y. (2023). Molecular Mechanism of Casein-Chitosan Fouling during Microfiltration. Sep. Purif. Technol..

[37] Jamshidian H., Rafe A. (2024). Complex Coacervate of Wheat Germ Protein/High Methoxy Pectin in Encapsulation of d-Limonene. Chem. Biol. Technol. Agric..

[38] Schmidt I., Cousin F., Huchon C., Boué F., Axelos M.A.V. (2009). Spatial Structure and Composition of Polysaccharide−Protein Complexes from Small Angle Neutron Scattering. Biomacromolecules.

[39] Liew M.K.H., Fane A.G., Rogers P.L. (1997). Fouling of Microfiltration Membranes by Broth-Free Antifoam Agents. Biotechnol. Bioeng..

[40] Wang J., Liu Z., Zheng K., Yuan Z., Yang C. (2024). Effect of pH on the Formation Mechanisms, Emulsifying Properties and Curcumin Encapsulation of Oat Protein Isolate–High Methoxy Pectin Complexes. Food Hydrocoll..

[41] Chiao Y.-H., Chen S.-T., Sivakumar M., Ang M.B.M.Y., Patra T., Almodovar J., Wickramasinghe S.R., Hung W.-S., Lai J.-Y. (2020). Zwitterionic Polymer Brush Grafted on Polyvinylidene Difluoride Membrane Promoting Enhanced Ultrafiltration Performance with Augmented Antifouling Property. Polymers.

[42] Zhi C., Xu J., Chen Y., Dong L., Bai Y., Zhang C. (2025). Reinforced-Concrete Inspired Porous Polymeric Membranes: Improved Mechanical Robust and Compaction Resistance via Incorporating Cellulose Nanofibers. J. Appl. Polym. Sci..

[43] Conidi C., Drioli E., Cassano A. (2020). Perspective of Membrane Technology in Pomegranate Juice Processing: A Review. Foods.

[44] Heidebrecht H.-J., Toro-Sierra J., Kulozik U. (2018). Concentration of Immunoglobulins in Microfiltration Permeates of Skim Milk: Impact of Transmembrane Pressure and Temperature on the IgG Transmission Using Different Ceramic Membrane Types and Pore Sizes. Foods.

[45] Peng B., Li Z., Xiong Q., Wu C., Huang J., Zhou R., Jin Y. (2022). Casein-Dextran Complexes Subjected to Microfiltration: Colloidal Properties and Their Corresponding Processing Behaviors. J. Food Eng..

[46] Ng A.N.L., Kim A.S. (2007). A Mini-Review of Modeling Studies on Membrane Bioreactor (MBR) Treatment for Municipal Wastewaters. Desalination.

[47] Holopainen-Mantila U., Vanhatalo S., Lehtinen P., Sozer N. (2024). Oats as a Source of Nutritious Alternative Protein. J. Cereal Sci..

[48] Li M., Huang G., Chen X., Xu Z., Huang J., Yin J., Feng R., Chen N., Read S., Wang S. (2025). Development of an EOR-Produced Petroleum Wastewater Treatment System through Integrated Polyacrylonitrile Membrane and ZrO_2_/Sericin Technologies: Revelation of Interactive Mechanism Based on Synchrotron and XDLVO Analyses. Npj Clean Water.

