# Supplementary material for: Membrane Fouling Mechanisms in the Microfiltration of Oat Protein–β-Glucan Complexes
Source: Membranes (Basel). 2026 Mar 27;16(4):116. doi: 10.3390/membranes16040116 (PMC13117436; doi:10.3390/membranes16040116)
Supplement: Supplementary file 1 [file membranes-16-00116-s001.zip › membranes-4191713-supplementary.pdf]

membranes

MDPI

Supplementary Materials

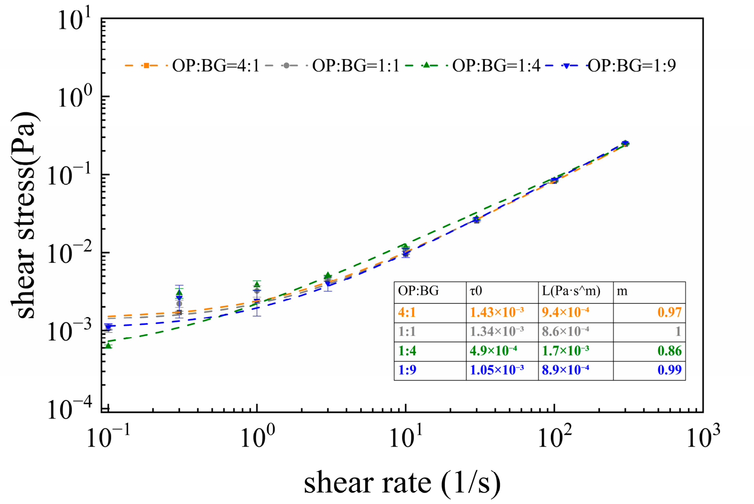

Figure S1. Rheological curves of oat protein–β-glucan complexes in the conditions of different oat protein–β-glucan ratios.

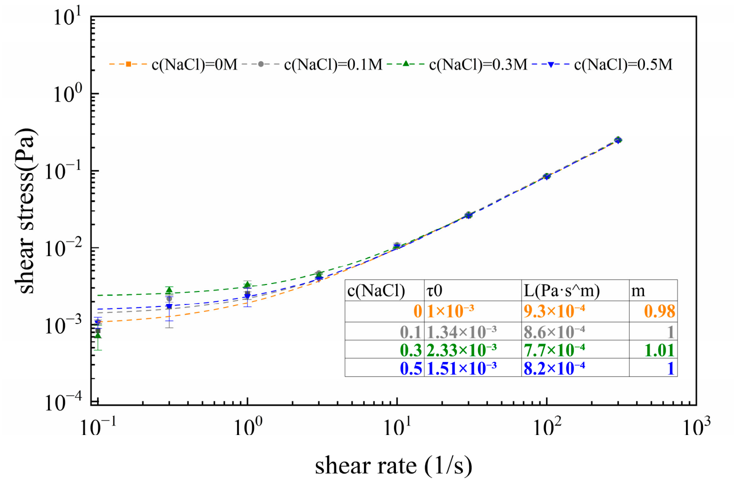

Figure S2. Rheological curves of oat protein–β-glucan complexes in the conditions of different sodium ion concentrations.

 https://doi.org/10.3390/membranes16040116

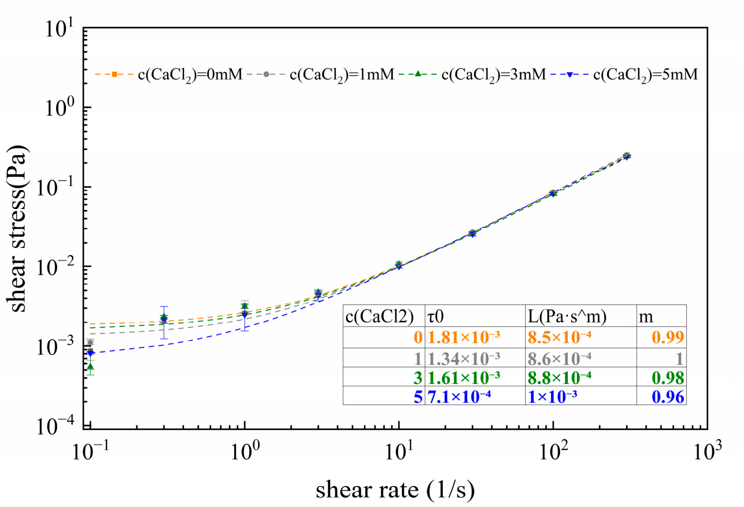

Figure S3. Rheological curves of oat protein–β-glucan complexes in the conditions of different calcium ion concentrations.

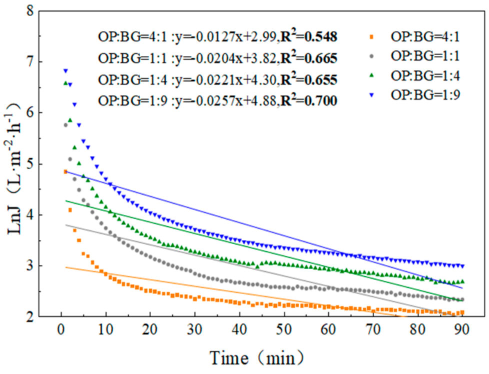

(A)

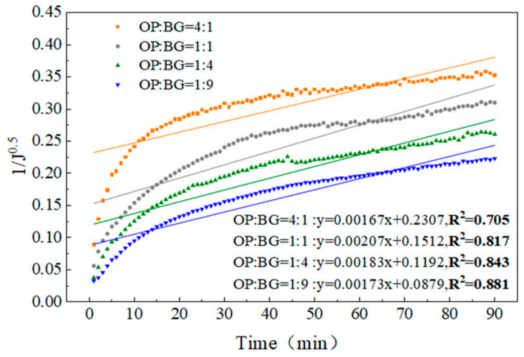

(B)

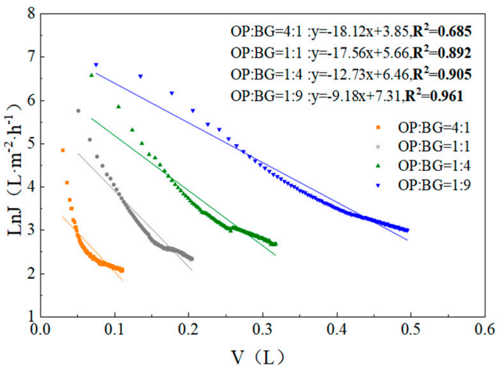

(C)

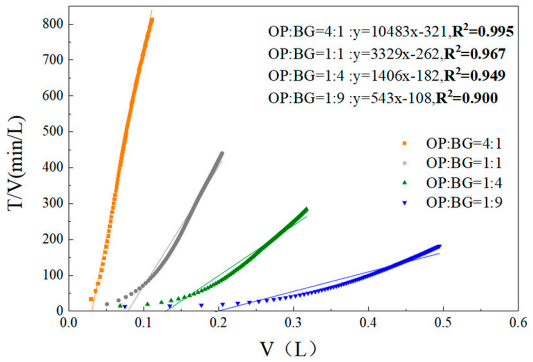

(D)

Figure S4. Pore blocking models fitting of oat protein–β-glucan complexes microfiltration with different oat protein–β-glucan ratios. A,B,C,D represent complete blocking,standard blocking,intermediate blocking and cake filtration respectively.

https://doi.org/10.3390/membranes16040116

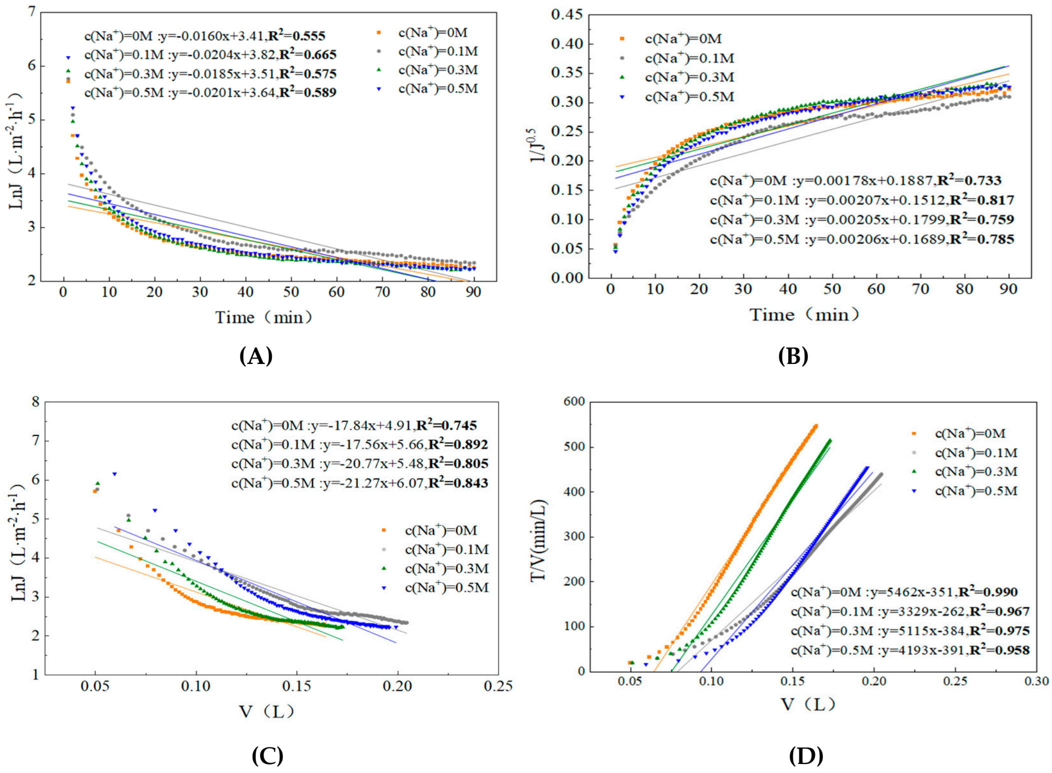

Figure S5. Pore blocking models fitting of oat protein–β-glucan complexes microfiltration with different sodium ion concentrations. A,B,C,D represent complete blocking,standard blocking,intermediate blocking and cake filtration respectively.

https://doi.org/10.3390/membranes16040116

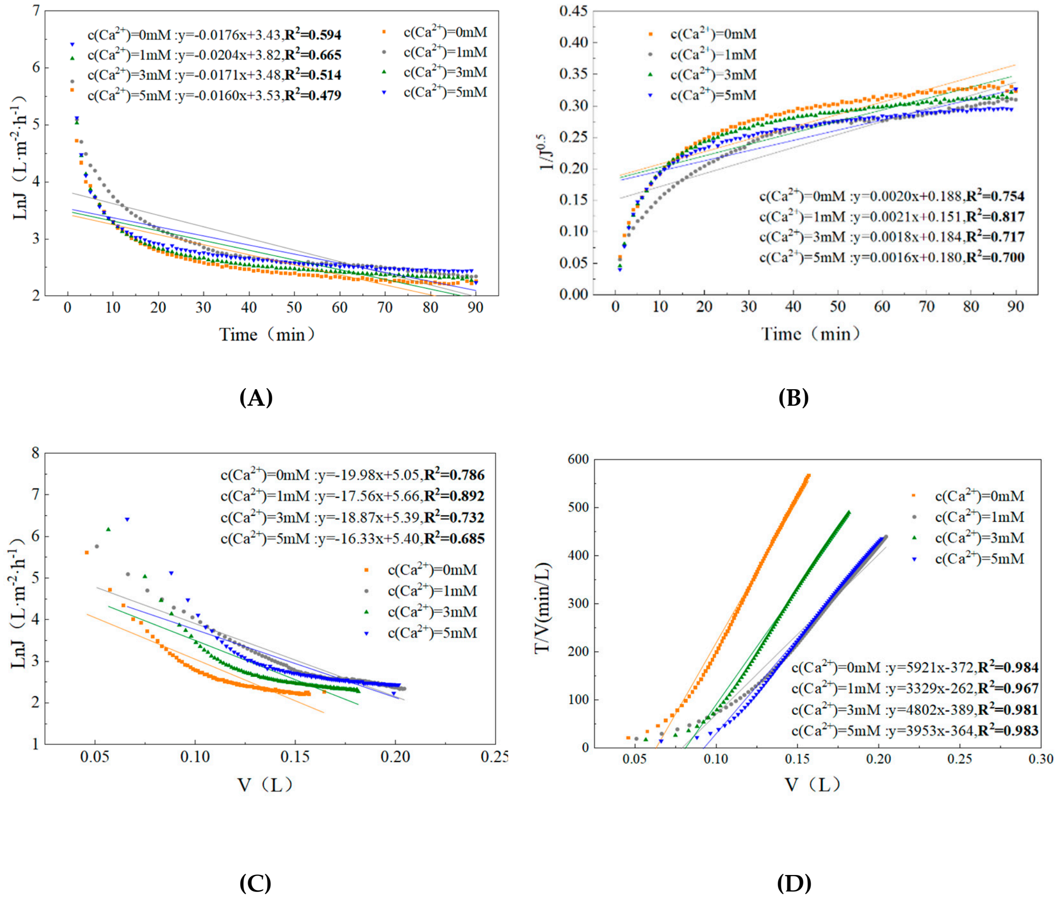

Figure S6. Pore blocking models fitting of oat protein–β-glucan complexes microfiltration with different calcium ion concentrations. A,B,C,D represent complete blocking,standard blocking,intermediate blocking and cake filtration respectively.

Disclaimer/Publisher's Note: The statements, opinions and data contained in all publications are solely those of the individual author(s) and contributor(s) and not of MDPI and/or the editor(s). MDPI and/or the editor(s) disclaim responsibility for any injury to people or property resulting from any ideas, methods, instructions or products referred to in the content.
